# Suppression of extracellular invertase inhibitor gene expression improves seed weight in soybean (*Glycine max*)

**DOI:** 10.1093/jxb/erw425

**Published:** 2016-12-07

**Authors:** Xiaofei Tang, Tao Su, Mei Han, Lai Wei, Weiwei Wang, Zhiyuan Yu, Yongguo Xue, Hongbin Wei, Yejie Du, Steffen Greiner, Thomas Rausch, Lijun Liu

**Affiliations:** 1 Soybean Research Institute, Academy of Agricultural Sciences, Harbin, 150086, China; 2 Plant Molecular Physiology, Centre for Organismal Studies (COS), Heidelberg University, Im Neuenheimer Feld 360, Heidelberg 69120, Germany; 3 Co-Innovation Center for Sustainable Forestry in Southern China, College of Biology and Environment, Nanjing Forestry University, Nanjing 210037, China

**Keywords:** Cell wall invertase, *Glycine max*, invertase inhibitor, post-translational regulation, seed weight, source and sink.

## Abstract

Cell wall invertase (CWI) and vacuolar invertase (VI) play multiple functions in plant growth. As well as depending on transcriptional and post-transcriptional regulation, there is growing evidence that CWI and VI are also subject to post-translational control by small inhibitory proteins. Despite the significance of this, genes encoding inhibitors, their molecular and biochemical properties, and their potential roles in regulating seed production have not been well documented in soybean (*Glycine max*). In this study, two invertase inhibitor isoforms, GmCIF1 and GmC/VIF2, were characterized to possess inhibitory activities *in vitro* via heterologous expression. Transcript analyses showed that they were predominantly expressed in developing seeds and in response to ABA. In accordance with this, surveys of primary targets showed subcellular localizations to the apoplast in tobacco epidermis after expressing YFP-fusion constructs. Investigations using RNAi transgenic plants demonstrated marked elevations of CWI activities and improvements in seed weight in conjunction with higher accumulations of hexoses, starch, and protein in mature seeds. Further co-expression analyses of *GmCIF1* with several putative CWI genes corroborated the notion that GmCIF1 modulation of CWI that affects seed weight is mainly contingent on post-translational mechanisms. Overall, the results suggest that post-translational elevation of CWI by silencing of *GmCIF1* expression orchestrates the process of seed maturation through fine-tuning sucrose metabolism and sink strength.

## Highlight

The roles of GmCIF1 and GmC/VIF1 as invertase inhibitors were examined *in vitro* in soybean (*Glycine max*), and post-translational elevation of extracellular invertase activity by silencing *GmCIF1* expression was found to increase seed weight.

## Introduction

Invertases (EC 3.2.1.26) irreversibly catalyze the hydrolysis of sucrose into hexoses (glucose and fructose), both of which are basic nutrients and signaling molecules in plants (Koch, 2004; Rolland *et al.*, 2006). Based on their pH optima and subcellular localizations, invertases are categorized into cytosolic neutral/alkaline invertase (CI) and acid invertase. These two types of invertases differ substantially in amino acid sequences and biochemical properties (Sturm, 1999). Acid invertases are further classified into cell wall invertase (CWI) and vacuolar invertase (VI) on the basis of their cellular targets. Unlike acid invertases, CIs are not glycosylated proteins and exhibit little sequence homology with CWIs and VIs (Ruan *et al.*, 2010).

CWIs and VIs are recognized as essential players in sugar metabolism and sugar signaling, thereby affecting source–sink interactions and responses to environmental cues in higher plants (Ruan, 2014). The roles of VI in the major regulation of carbon metabolism, hexose distribution, cellular osmolarity, and oxidative stress defense have been extensively explored (Kohorn *et al.*, 2006; Sergeeva *et al.*, 2006; Nägele *et al.*, 2010; Xiang *et al.*, 2011; Liu *et al.*, 2015). Suppression of VI activities alters soluble sugar conversion, thereby decreasing cold-induced sweetening in potato tubers (Bagnaresi *et al.*, 2008; Bhaskar *et al.*, 2010; Zhu *et al.*, 2016) and affecting fruit size (Klann *et al.*, 1996; Tang *et al.*, 1999; Yu *et al.*, 2008). In contrast, CWIs catalyze the cleavage of sucrose into hexoses, which are unloaded into sink cells of the apoplast by sucrose transporters (SUTs) from the phloem (Bihmidine *et al.*, 2013). The hexoses released in sink tissues may go either into intracellular compartments for metabolism, polysaccharide synthesis, and gene regulation, or into extracellular compartments for fungal colonization and defense-related responses (Roitsch *et al.*, 2003; Doidy *et al.*, 2012). It is therefore not surprising that CWIs play multifaceted and significant roles in response to environmental stimuli and have a major influence on the development of sink organs (Rausch and Greiner, 2004; Proels and Hückelhoven, 2014). Overexpression of CWI genes delays leaf senescence and increases drought resistances in tobacco and tomato (Lara *et al.*, 2004; Albacete *et al.*, 2015). In Arabidopsis, CWI is considered to be an essential component of pathogen-induced plant defense (Zhao *et al.*, 2015). *AtCWI4* has been shown to be associated with floral nectar secretion (Ruhlmann *et al.*, 2010). Ectopic expression of CWIs in Arabidopsis improves plant vegetative and reproductive growth (Heyer *et al.*, 2004; von Schweinichen and Büttner, 2005). A null mutant of the CWI gene in maize, *Mn1*, led to miniature seeds as a result of the arrested endosperm development (Miller and Chourey 1992), whereas overexpression of *Mn1* increased grain yield and starch content (Li *et al.*, 2013). The critical roles of CWIs in regulation of seed development have also been well documented in rice, tomato, and cotton (Wang *et al.*, 2008; Zanor *et al.*, 2009; Wang and Ruan, 2012), confirming that CWIs exert multiple signaling and metabolic functions in plants.

Earlier research on the regulation of acid invertases primarily focused on transcriptional and post-transcriptional control via increases in their transcripts in response to diverse signals (Weber *et al.*, 1996; Ehneß and Roitsch, 1997; Roitsch, 1999). However, CWI and VI are intrinsically stable proteins owing to their glycan decoration, and hence the regulation of CWI and VI may also be subject to post-translational mechanisms. An increasing body of *in vitro* studies have shown that CWI and VI activities are determined by small inhibitor of β-fructosidases (IFs), with sizes ranging from 15–23 kDa, with targets in the cell wall or vacuoles (Rausch and Greiner, 2004). The first putative invertase inhibitor was biochemically characterized in potato (Schwimmer *et al.*, 1961). Thereafter *in vivo* inhibitory functions of cell wall invertase inhibitors (CIFs) were validated in tobacco (Greiner *et al.*, 1998), followed by the identification of several CIFs in maize and tomato (Bate *et al.*, 2004; Reca *et al.*, 2008). Studies on the functional roles of vacuolar invertase inhibitors (VIFs) demonstrated a marked reduction of cold-induced sweetening in potato tubers by capping the activities of VIs (Greiner *et al.*, 1999; Brummell *et al.*, 2011; Liu *et al.*, 2013; Mckenzie *et al.*, 2013). More recently, silencing of a tomato CIF led to a delay of leaf senescence and improvements in seed filling and fruit size (Jin *et al.*, 2009). In Arabidopsis, suppression of two invertase inhibitors resulted in an increase of seed production (Su *et al.*, 2016), reflecting the fact that post-translational modulation of CWI is required for sugar unloading to sink tissues.

*Glycine max* (soybean), a legume crop, is an important source of edible proteins and oils that are widely used throughout the world (Song *et al.*, 2013). There has been ongoing interest in improving soybean performance, although significantly enhancing seed production remains a major challenge for modern agriculture. In spite of the advances made in commonly studied plants, little is known about invertase inhibitory proteins and their physiological significance in soybean. To our knowledge, there have been no reports on the targets of invertase inhibitors, on the post-translational regulation of CWI, or on the phenotypes that arise from modification of the expression of inhibitors in soybean plants. In a bid to rectify this situation, two putative soybean invertase inhibitors, GmCIF1 and GmC/VIF2, were functionally characterized by the use of recombinant proteins. Their subcellular targets were examined by ectopic expression with fusions of fluorescent protein. The functional role of GmCIF1 was further explored in RNAi transgenic plants via the silencing of its expression. Based on transcript and metabolic analysis, our work demonstrates the physiological roles of GmCIF1 in controlling seed maturation through specifically depressing CWI activities. The substantially improved seed weight reported here provides a promising strategy for future development, and in the long term, it may facilitate increases in crop production via manipulation of the post-translational modulation of CWI.

## Materials and methods

### Plant materials and growth conditions

Soybean cultivar ‘Heinong 53’ (HN53) was grown in standard potting soil in a greenhouse or in growth chambers with a temperature cycling between 18 °C (night) and 24 °C (day) with 55% relative humidity under long-day (16 h light/8 h dark, 300–400 μE) conditions. In all comparative analyses of tissues, wild-type and RNAi transgenic plants were cultivated and harvested simultaneously. Tobacco (*Nicotiana benthamiana*) plants were maintained in a growth chamber at 25 °C under a light regime of 16 h and 300 μE.

### Construction of RNA interference (RNAi) vector

A 430-bp fragment of fatty acid desaturation 2 (FAD2) intron I was amplified by using a forward primer FAD2I-F1 containing SmaI restriction sites, and a reverse primer FAD2I-R1 containing KpnI restriction sites. This PCR product was cloned into the pUC8 vector to generate pUC8-FAD2I. To form the typical RNAi structure, reverted repeat sequences of GmCIF1 (362 bp) applied in hairpin construction were amplified by using two different sets of primers: a forward primer GmCIF1-F-P1, containing the BamHI site, paired with a reverse primer GmCIF1-F-R1, containing an SmaI site, and a forward primer GmCIF1-R-P1, containing the KpnI site, paired with a reverse primer GmCIF1-R-R1, containing an SacI site. The two amplified target fragments were cloned into the pUC8-FAD2I vector to create the hairpin cassette. The hairpin cassette of GmCIF1-F/FAD2I/GmCIF1-R was further ligated into the vector pCAMBIA3300 (http://www.cambia.org/daisy/cambia/2070.html), which was used for soybean transformation (see Supplementary Fig. S3B at *JXB* online). The primer sequences are listed in Supplementary Table S2.

### Plant transformation

*Agrobacterium*-mediated transformation of soybean using the *bar* gene as a selectable marker has been described previously (Zeng *et al.*, 2004). Shoot initiation and elongation were primarily screened by the addition of the herbicide glufosinate to B5 basal medium (Gamborg *et al.*, 1968) in a Petri dish. The T3 homozygous generations were used for analysis. For transient transformation, *Agrobacterium tumefaciens* (*C58C1*) cells containing the appropriate construct were grown overnight in 30 ml of YEB-medium supplemented with carbenicillin (50 μg ml^–1^), rifampicin (100 μg ml^–1^) and spectinomycin (50 μg ml^–1^) until the stationary phase. After centrifugation at 3000 *g* for 30 min at ambient temperature, the cells were re-suspended in 10–15 ml of infiltration buffer [10 mM 2-(N-morpholino) ethanesulfonic acid (MES), pH 5.9, 150 μM acetosyringone] and incubated with gentle agitation for 2 h at room temperature. The cell suspensions were adjusted to an OD_600_ of 1.0 by mixing with infiltration buffer. As infiltration in soybean leaves did not work properly, the lower epidermis of 4- to 5-week-old tobacco leaves were transformed by infiltrating *Agrobacterium* cells harboring appropriate plasmids via a needleless syringe. After 40 h, the infiltrated regions were analyzed by confocal laser scanning microscopy (CLSM).

### Purification of recombinant GmCIF1 and GmC/VIF2

Coding sequences (omitting the signal peptide) of GmCIF1 and GmC/VIF2 were amplified from flowers using primers containing the GATEWAY (Invitrogen) cloning *attB1* and *attB2* sites and the TEV protease site (see Supplementary Table S2). PCR products were then recovered using a PCR purification kit (Thermo Scientific), sequenced, and inserted into the pDONR201 plasmid (Invitrogen) and subsequently recombined with the destination vector pETG-20A, which produced 6xHis-tagged thioredoxin A (TrxA) fusion constructs. The protein purifications were performed as described previously (Hothorn *et al.*, 2003; Link *et al.*, 2004). The *E. coli* strain Rosetta-gami^TM^ (DE3) (Novagen) was used as host for the protein expression. Bacteria were grown to a density of OD_600_ of 0.6 in lysogeny broth (LB) medium, followed by the addition of IPTG to 0.2 mM and further grown for 24 h at 18 °C. Cells were pelleted by centrifugation at 10 000 *g* for 15 min and extracted with 1/20 volume of lysis buffer (50 mM Na_2_HPO_4_/NaH_2_PO_4_, pH 7.0, 500 mM NaCl, 1% Triton X-100, 1 mg ml^–1^ lysozyme). After centrifugation at 15 000 *g* for 1 h, the supernatant was mixed with 0.6 g Ni-TED Protino resin (Macherey-Nagel) and stirred at 4 °C for 45 min before loading into a column. The column was washed with lysis buffer and washing buffer (50 mM Na_2_HPO_4_/NaH_2_PO_4_, pH 7.0, 500 mM NaCl, 10% glycerol), and bound fusion protein was then eluted with 10 volumes of the washing buffer containing 250 mM imidazole, and dialyzed against TEV protease cleavage buffer (50 mM Na_2_HPO_4_/NaH_2_PO_4_, pH 7.0, 200 mM NaCl). The eluted fractions were then cleaved with recombinant TEV protease for 3 h at 30 °C. The untagged inhibitor proteins were finally purified with a second elution.

### Functional assays of recombinant GmCIF1 and GmC/VIF2

To test the the inhibitory activities of recombinant inhibitors against acid invertase, variable amounts of recombinant inhibitory proteins were added to a suitable invertase preparation (see below) in assay buffer in a total amount of 200 µl and incubated for 30 min at 37 °C to allow complex formation, followed by the addition of 100 µl sucrose (100 mM) and incubated for 60 min at 37 °C. The reaction was terminated by the addition of sodium phosphate buffer (1 M, pH 7.5) and heating at 95 °C for 5 min. The amount of glucose released was determined as described below. In each experiment, samples without recombinant inhibitor proteins were included to determine background absorption, which was subtracted from the other reactions.

### Determination of acid invertase activity *in vitro*

The *in vitro* analysis of invertase activity was performed as described previously by Link *et al.* (2004). Selected tissues of soybean were ground in liquid nitrogen using a TissueLyser (Qiagen) and homogenized in 500 μl extraction buffer (30 mM MOPS, 250 mM sorbitol, 10 mM MgCl2, 10 mM KCl, and 1 mM PMSF, pH 6.0). After centrifugation (10 min, 8500 *g*, 4 °C), the pellets were washed once with extraction buffer plus 1% Triton X-100, and twice with extraction buffer only. The cell-wall pellets were re-suspended in 500 μl assay buffer (20 mM triethanolamine, 7 mM citric acid, and 1 mM PMSF, pH 4.6), and used for the determination of CWI activity. For VI extraction, endogenous sucrose was removed by acetone precipitation of the soluble fraction with four volumes of ice-cold acetone (20 min, −20 °C). After centrifugation (10 min, 15 000 *g*, 4 °C), the pellets were re-suspended in one volume of assay buffer.

For the measurement of enzyme activity, 100 μl of the obtained preparations were incubated with 100 μl sucrose (100 mM) and deionized water up to 300 μl. After incubation for 1 h at 37 °C, the reaction was stopped by the addition of 30 μl sodium phosphate (1 M, pH 7.5) and heating at 95 °C for 5 min. Assay were performed in quadruplicate, one of which was neutralized and boiled immediately after sucrose addition. The activity of this sample was subtracted from the activity of the others as background absorption. Liberated glucose was measured in a coupled enzymatic–optical assay. In this assay, 100 μl of the reaction mixture, 20 μl 30 mM ATP, 20 μl 30 mM NADP, 2 μl Hexokinase/Glucose-6-phosphate dehydrogenase suspension (340 U ml^–1^ HK, 170 U ml^–1^ G6P-DH, Roche) and up to 1 ml buffer (40 mM triethanolamine, 8 mM MgSO4, pH 7.5) were mixed and incubated for 5 min at room temperature. Formation of NADPH was measured spectrophotometrically at 340 nm and the liberated glucose was calculated according to the Lambert–Beer Law. Invertase activity was expressed in nkat g^–1^ fresh weight (1 nkat = 1 nmole glucose liberated per second).

### Expression analysis

RNA extraction and cDNA synthesis were performed as described previously by Han *et al.* (2013). Selected tissues of *Glycine max* were ground in liquid nitrogen using a TissueLyser (Qiagen). Total RNA was extracted by using the Gene MATRIX Universal RNA purification Kit (Roboklon) according to the manufacturer’s instructions. RNA quality was determined by NanoDrop 2000 (Thermo Scientific). For a standard qRT-PCR technical application, the reactions were prepared in a 15 μl volume, containing 5 μl cDNA sample (appropriately diluted), primers, dNTP, SYBR green, and Jumpstart Taq-DNA polymerase (Sigma). The mixture was subjected to a temperature regime of 95 °C for 6 min, followed by 95 °C for 20 s, 58 °C for 20 s, and 72 °C for 20 s for 40 cycles, followed by a melt cycle from 65 °C to 95 °C. Reactions were run on a Stratagene Mx3000p QPCR Systems (Thermo Scientific). The efficiency of the primers was tested in preliminary experiments with dilutions of cDNA, producing an *R*^2^ value ≥0.99. The relative expression level of a target gene was calculated by normalizing to the geometric mean (Vandesompele *et al.*, 2002) of multiple reference genes: *EF/αb* (*EV279336*), *CYP* (*CF806591*), *Actin2*/7 (*BW677100*), and *ActinII* (*BW652479*). The primer sequences are listed in Supplementary Table S2. Each experiment had three biological repeats, each with three technical replicates.

Transcriptome sequencing (RNA-seq) analysis of tissue-specific expressions of the soybean CWI genes was performed as described previously by Severin *et al*, (2010). Gene expression levels were estimated using the values of number of mapped reads per kilobase of the exon region per million mapped reads (RPKM). Data contained within the RNA-seq is publically available at the SoyBase database (http://www.soybase.org/soyseq/).

### Subcellular localization of GmCIF1 and GmC/VIF2

The coding sequences (omitting the stop codon) of *GmCIF1* and *GmC/VIF2* were amplified by PCR, including the primers containing the Gateway (Invitrogen) *attB1* and *attB2* recombinant sites (see Supplementary Table S2). The PCR products were recovered by using the GeneJET PCR purification kit (Thermo Scientific) and then inserted into the pDONR201 donor plasmid, sequenced, and subsequently recombined with the binary destination vector pB7YWG2.0, yielding the pB7GmCIF1-YFP and pB7GmC/VIF2-YFP constructs. Tobacco leaves were co-infiltrated with *A. tumefaciens* (*C58C1*) harboring pB7GmCIF1-YFP (or pB7GmC/VIF2-YFP) and a cell wall-localization marker pK7BvCWI-1RFP (Rosenkranz *et al.*, 2001). The infiltrated region of the leaves was analyzed by CLSM. Images were taken with a Zeiss LSM 510 Meta inverted microscope. The yellow fluorescent protein (YFP) was excited with a 514-nm laser line, and the emitted fluorescence was collected using a 530–600-nm band pass filter. The red fluorescent protein (RFP) was excited with 543-nm laser line and the emitted fluorescence was collected with a 560-nm long pass filter.

### Quantification of sugars, protein and oil contents

Soluble sugars were extracted from 100 mg of homogenized seeds with 900 μl of 80% ethanol for 1 h at 80 °C, and then centrifuged at 12 000 *g* for 10 min at 4 °C. The collected supernatant was dried in a vacuum overnight. The sugar content of 350 μl of the re-dissolved solution was quantified by high-performance anion-exchange chromatography with pulsed 25 amperometric detection (HPAEC-PAD) in an ICS-3000 system (Dionex) with a CarboPac PA1 column and 15–300 mM NaOH (Fluka) in HPLC-water (VWR) as the eluent. Quantitative calculation of sugars was performed using the Chromeleon software 6.7 (Dionex). The final pellets were used for the quantification of starch content after degradation with amyloglucosidase and α-amylase, as described by Focks and Benning (1998).

Protein and oil contents in soybean seeds were determined by near-infrared spectroscopy (MATRIX-I, Bruker) with a near-infrared grain analyser as described by Delwiche (1998).

### ABA treatment

Plants at 40 d after germination (DAG) were sprayed with 50 µmol ABA (dissolved in 10% ethanol) or 10% ethanol (control) once a day for 4 d. Mature leaves were harvested for RNA isolation and assessment of acid invertase activities after 5 d growing in the greenhouse.

### Germination test

Seeds (60–80) of wild-type and RNAi plants were surface-sterilized with 70% ethanol, and either rinsed with water for fresh use or with 100% ethanol and air-dried. Germination tests were carried out in Petri dishes with 4 layers of autoclaved moist filter paper. After a 4-d stratification period at 4 °C in the dark, the dishes were incubated in long-day conditions of 16 h/8 h light/dark at 24/18 °C. Germination was scored by radicle emergence from the seed coat. The germination rate was documented daily for 2–5 d.

### Accession numbers

Sequence data can be found in the GenBank database (https://www.ncbi.nlm.nih.gov/genbank/) under the following accession numbers: NtCIF (Y12805); NtVIF (AY145781); AtCIF1 (AT1G47960); AtC/VIF2 (AT5G64620); BvC/VIF (XP_010685378); IbC/VIF (AAM94391); CiC/VIF (KVH90191); SlCIF1 (CAA09420); SlINVINH1 (AJ010943); GmCIF1 (BT090960); GmC/VIF2 (BT091584); StINVINH1 (GU321338); StINVINH2A (GU321341); StINVINH2B (GU321342); ZmINVINNH1 (AATT24363).

## Results

### Cloning and characterization of *GmCIF1* and *GmC/VIF2*

**Fig. 1.**
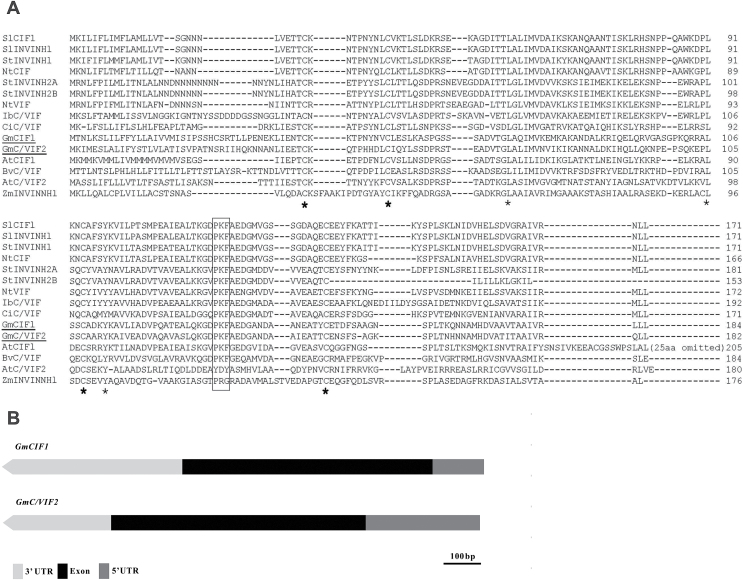
Multiple sequence alignment of functionally characterized invertase inhibitors and gene structures. (A) Protein sequence alignment was conducted using Clustal Omega (http://www.ebi.ac.uk/Tools/msa/clustalo/). Four cysteine residues (C) are labeled with bold asterisks, and the conserved motif is indicated by a box. Nt, *Nicotiana tabacum*; At, *Arabidopsis thaliana*; Bv, *Beta vulgaris*; Ib, *Ipomoea batatas*; Ci, *Cichorium intybus*; Sl, *Solanum lycopersicum*; Gm, *Glycine max*; St, *Solanum tuberosum*; Zm, *Zea mays*. (B) *GmCIF1* and *GmC/VIF2* showing a similar genomic structure.

Systematic BLAST searches within the Genbank database retrieved a large number of *C/VIF*-like genes in the soybean genome. Two of them, *GmCIF1* (BT090960) and *GmC/VIF2* (BT091584), encoding putative invertase inhibitors were identified with high homologies (>40% amino acid identity) to *AtCIF1* from Arabidopsis (Link *et al.*, 2004; Su *et al.*, 2016). A further survey of protein sequences by performing a BLAST search against the SoyBase database (http://www.soybase.org/) confirmed that GmCIF1 and GmC/VIF2 were encoded by Glyma.17G036300 and Glyma.17G036400, respectively. These two isoforms have a similar genomic structure and protein size, sharing 54% amino acid identity (Fig. 1A, B). After removal of the N-terminal signal peptides, the deduced mature proteins of GmCIF1 and GmC/VIF2 are comprised of 155 and 157 amino acid residues, respectively. The predicted molecular weight is 16.77 kDa for GmCIF1, a pI of 6.06, and 16.72 kDa for GmC/VIF2, a pI of 7.59. Protein sequence alignment with other known C/VIFs (Fig.1A) showed that GmCIF1 and GmC/VIF2 contain typical hallmarks, namely four conserved cysteine (C) residues that form two disulfide bridges to sustain protein stability, and a conserved motif (PKF) that has been demonstrated by crystallographic analysis to interact for inhibitory functions with the invertase substrate cleft (Hothorn *et al.*, 2010). Phylogenetic analyses showed that GmCIF1 and GmC/VIF2 are clustered into a clade together with an experimentally determined AtCIF1 from Arabidopsis, whereas their locations were far away from the C/VIFs in tomato, potato, and tobacco (see Supplementary Fig. S1).

### Heterologous expression and inhibitory functions of GmCIF1 and GmC/VIF2

To verify their inhibitory functions *in vitro*, the coding sequences of GmCIF1 and GmC/VIF2 were amplified with cDNA prepared from flowers. Sequences encoding mature proteins (omitting the signal peptides) were cloned into the pETG-20A vector (see Supplementary Fig. S2A). Heterologous expression in the *E. coli* strain Rosetta-gami™ (DE3) yielded N-terminal TrxA fusion proteins for GmCIF1 and GmC/VIF2. Under native conditions, the fusion proteins were released by the cleavage of the TEV protease. The purified GmCIF1 and GmC/VIF2 were recovered by Ni-TED affinity chromatography to remove the His-tagged TrxA and TEV protease (Supplementary Fig. S2B). Processes during the protein purification suggested that recombinant GmCIF1 and GmC/VIF2 were soluble. The sizes of both the finally purified proteins were close to the predicted molecular weight. Further analysis of their mobility on SDS-PAGE under non-reducing conditions revealed increased shifts, reflecting the presence of active intramolecular disulfide bridges in the recombinant proteins (data not shown).

**Fig. 2.**
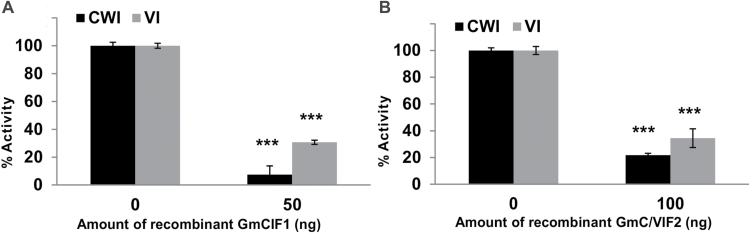
The *in vitro* inhibitory effects of the recombinant GmCIF1 and GmC/VIF2. (A) The lowest dose (50 ng) of GmCIF1 caused maxmium suppression of activities of CWI and VI. (B) The lowest dose (100 ng) of GmC/VIF2 caused maxmium suppression of activities of CWI and VI. Mature soybean leaves were harvested for the extraction of CWI and VI. The enzyme activity data represent means ±SE of at least four independent biological replicates and asterisks indicate significant differences in comparison with the control using Student’s *t*-test: ***P<0.001.

Inhibitory activities of the recombinant proteins were determined by incubating with acid invertase fractions that were extracted from leaves. Approximate 50 ng recombinant GmCIF1 was demonstrated to exert the maximum inhibitory effects and led to a significant depression of 95% in CWI activity and 70% in VI activity (Fig. 2A). Both CWI and VI were increasingly inhibited by increasing amounts of the recombinant GmCIF1 (see Supplementary Fig. S2C, D). However, incubating with even higher amounts (100 ng and 1000 ng) of the recombinant GmCIF1 did not affect the activities of CWI and VI further. In comparison with GmCIF1, the use of approximately 100 ng of the recombinant GmC/VIF2 resulted in maximum inhibition, causing an 80% decrease in CWI activity and a 75% decrease in VI activity (Fig. 2B). It is noteworthy that both recombinant GmCIF1 and GmC/VIF2 exhibited higher inhibitory affinities to CWI than VI *in vivo*, suggesting their putative roles as CIFs *in vivo*.

### Expression patterns of *GmCIF1* and *GmC/VIF2*

**Fig. 3.**
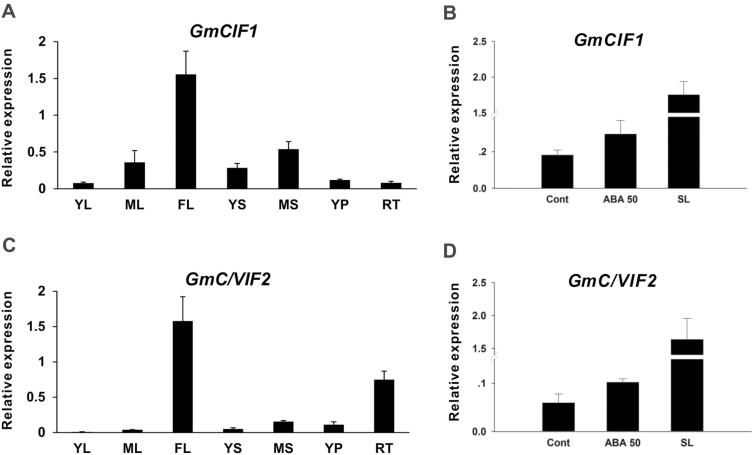
Expression analyses of *GmCIF1* and *GmC/VIF2*. (A, C) Transcript levels of *GmCIF1* and *GmC/VIF2* in different soybean tissues. (B, D) Expression of *GmCIF1* and *GmC/VIF2* in response to ABA and ABA-triggered leaf senescence. Data represent mean values ±SE of at least three independent biological replicates for qRT-PCR. *GmACT2/7*, *GmACTII*, *GmEF/αb*, and *GmCYP* were used as reference genes. YL, young leaf; ML, mature leaf; FL, flower; YS, seed 15 d after flowering (DAF); MS, seed 25 DAF; YP, young pods 10 DAF; RT, root; Cont, control; ABA 50, 50 µM ABA; SL, senescent leaves induced with 200 µM ABA.

To analyze the expression characteristics of *GmCIF1* and *GmC/VIF2*, their transcripts were investigated by qRT-PCR. The tissue-specific expression patterns were examined in seven tissues (young leaf, mature leaf, flower, young seed, mature seed, young pod, and root). The results showed that *GmCIF1* and *GmC/VIF2* displayed similar expression profiles; however, the transcript accumulation of *GmCIF1* was more abundant than *GmC/VIF2* (Fig. 3A, C). Both of them exhibited higher transcript levels in flowers and developing seeds. *GmCIF1* was also observed to be expressed more in mature leaves than in young leaves (Fig. 3A), whilst *GmC/VIF2* showed a relatively high expression level in roots (Fig. 3C). To test whether their transcripts are promoted in response to ABA and ABA-triggered leaf senescence, different concentrations of ABA were sprayed onto mature leaves. It was found that expression of *GmCIF1* and *GmC/VIF2* both increased upon 50 µM ABA treatment (Fig. 3B, D). Notably, treating with a higher concentration of ABA (200 µM) led to an accelerated process of leaf ageing, whilst the transcripts of *GmCIF1* and *GmC/VIF2* were significantly increased (Fig. 3B, D), suggesting that both *GmCIF1* and *GmC/VIF2* are ABA- and senescence-responsive genes.

### GmCIF1 and GmC/VIF2 target to the apoplast

**Fig. 4.**
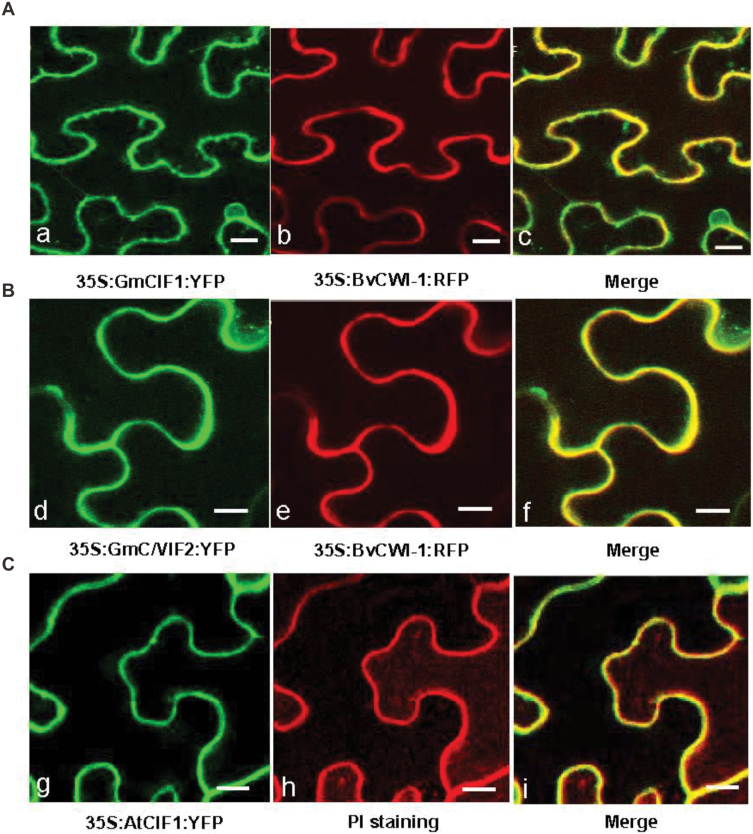
Apoplastic localization of GmCIF1 and GmC/VIF2 in tobacco leaves. Tobacco leaves were co-infiltrated with *Agrobacterium* culture harboring the constructs of (A) *35S:GmCIF1:YFP* and *35S:BvCWI-1:RFP* or (B) *35S:GmC/VIF2:YFP* and *35S: BvCWI-1:RFP*. (C) Apoplastic localization of AtCIF1 and PI (propidium iodide) staining. (a, d) Epidermal cells depicting YFP (green) fluorescence. (b, e) RFP (red) fluorescence in the cell wall. (c, f) Overlap of fluorescent signals (yellow) from YFP and RFP. A sugar beet CWI (*BvCWI-1*) was used as cell wall-localization marker.

To determine the primary target of GmCIF1 and GmC/VIF2 within cells, prediction programs were applied to deduce their intracellular compartmentations. All three *in silico* analyses of GmCIF1 and GmC/VIF2 unanimously resulted in the highest scores for an extracellular target (see Supplementary Table S1). We then sought to verify the subcellular localizations using fluorescent-labelled GmCIF1 and GmC/VIF2. The *35S:GmCIF1:YFP* and *35S:GmC/VIF2:YFP* C-terminal fusion constructs were co-delivered with a RFP-fused cell wall-localization marker (*35S:BvCWI-1:RFP*) into tobacco leaf epidermal cells by a transient ectopic expression. By co-infiltration, a yellow fluorescent signal was visualized around the cell periphery from the overlap of the YFP signal (green) and the RFP signal (red), indicating an apoplastic localization (Fig. 4A, B). However, there was no signal captured from vacuoles. Concurrently, as a comparison, the YFP-fused Arabidopsis extracellular invertase inhibitor AtCIF1 (*35S:AtCIF1:YFP*) was transformed into tobacco leaves. Similarly, combined with the propidium iodide (PI) staining, overlapped fluorescent signals (yellow) between PI (red) and YFP (green) suggested an apoplastic target (Fig. 4C). Taken together, the fluorescent images of the co-localization of AtCIF1 and PI further support the view that GmCIF1 and GmC/VIF2 are primarily targeted to the apoplast, which is in agreement with the computational prediction of their subcellular localizations.

### CWI activities are increased in transgenic RNAi plants

**Fig. 5.**
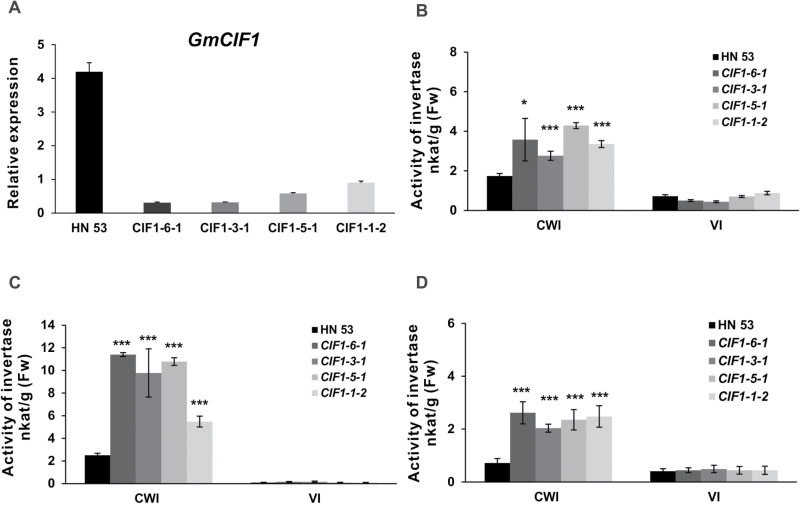
Effects on *GmCIF1* expression and acid invertase activities in RNAi plants. (A) Suppression of *GmCIF1* expression. (B) Increases of CWI activities in seeds at 45 d after flowering (DAF). (C) Increases of CWI activities in seeds at 65 DAF (dehydrated). (D) Increases of CWI activities in imbibed seeds (24 h). Data represent mean values ±SE of at least three independent biological replicates for qRT-PCR. *GmACT2/7*, *GmACTII*, *GmEF/αb*, and *GmCYP* were used as reference genes. The enzyme activity data represent means ±SE of at least four independent biological replicates and asterisks indicate significant differences in comparison with the control using Student’s t-test: ***P<0.001, *P<0.05.

To investigate the physiological roles of GmCIF1 and GmC/VIF2, transgenic soybeans were generated by a strategy of RNA interference (RNAi) to inhibit the expressions of *GmCIF1* and *GmC/VIF2*. Four out of 32 independent homozygous plants with silencing of *GmCIF1* were screened; however, no positive *GmC/VIF2*-silenced plants were identified owing to unsuccessful transformation. The selected *GmCIF1*-silenced plants displayed significantly decreased transcript levels of *GmCIF1*, ranging from 82% to 95%, in comparison with wild-type plants (Fig. 5A). To test whether these observations were due to the specific suppression of *GmCIF1* expression, we seeded a BLAST search for homologs of GmCIF1 by inputting the partial coding sequence that was used for the RNAi vector construction (see Supplementary Fig. S3A, B). The search in SoyBase retrieved two genes (Glyma.07G237300 and *GmC/VIF2*) that exhibited the highest scores of identities (>50%). However, transcript analyses of these genes showed no apparent expression differences between the RNAi plants and the wild-type control (Supplementary Fig. S3C), suggesting that the RNA-mediated gene silencing particularly targets to GmCIF1.

The observed tissue-specific expression profiles of *GmCIF1* prompted us to examine whether the acid invertase activities were differentially regulated in sources and sinks. In contrast to the wild-type control, analysis of enzyme activities in RNAi plants revealed that CWI activities significantly increased as seeds matured (Fig. 5B, C). In addition, significant increases of CWI activities were also detected early in seed germination (24 h) (Fig. 5D) and in mature leaves of RNAi plants (see Supplementary Fig. S4D). Particularly in dehydrated mature seeds (65 DAF), CWI activities of RNAi plants showed significantly higher levels than in wild-type plants, ranging from 1.5-fold to 4-fold higher (Fig. 5C). In contrast to CWI, VI activities appeared not to be significantly influenced as only minor changes were observed between RNAi plants and the wild-type control, except for at the early seed maturity stage (Supplementary Fig. S4A). VI activities declined to undetectable levels until the seed completed dehydration (65 DAF, Fig. 5C). Taken together, the results showed that in the RNAi transgenic soybean the silencing of *GmCIF1* expression led to significant elevations of CWI activities, suggesting that CWI is the physiological target of GmCIF1.

### Silencing of GmCIF1 significantly improves seed weight

**Fig. 6.**
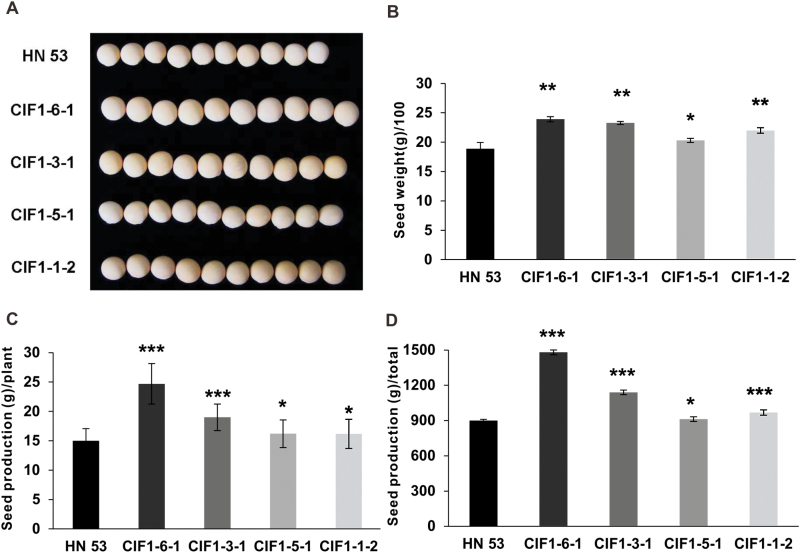
Phenotypic impacts on seed weight and production. (A) Image of seeds showing increases in size in RNAi plants (10 seeds are shown in each case). (B) Increases in the seed weight (100 seeds) in RNAi plants. (C) Seed production per individual plant increased in RNAi plants. (D) Total seed production increased in RNAi plants (40 plants). The data represent means ±SE of at least five biological replicates and asterisks indicate significant differences in comparison with the control using Student’s *t*-test: ***P<0.001, **P<0.01, *P<0.05.

Given that the silencing of the *GmCIF1* transcript increased CWI activities, we then explored whether there were any potential phenotypic changes in plant growth and development. Interestingly, the morphological analysis showed significant differences in seed weight between RNAi plants and the wild-type plants. RNAi plants produced larger seeds than the wild-type control (Fig. 6A, B; see Supplementary Fig. S5A). In the same backgrounds, analyses of the seed production per individual plant and the total across all plants showed a statistical improvement (Fig. 6C, D). However, even if very large increases in CWI activities were detected at the beginning of seed germination (24 h) of RNAi plants, the germination rate was not statistically affected (Supplementary Fig. S5B). RNAi plants appeared to grow as normally as the wild-type plants and no significant differences were recorded for the plant height in RNAi plants (data not shown). ABA perturbation caused marked depressions of activities of both CWI and VI in leaves, whereas CWI activities of RNAi plants remained at higher levels than the wild-type control plants (Supplementary Fig. S4D, E). However, RNAi plants and wild-type plants showed no differences in ABA-mediated leaf ageing (Supplementary Fig. S4B, C). Interestingly, in comparison with the wild-type control at 125 d, RNAi plants showed a prolonged plant greening period of approximately 4–6 d, which was statistically significant (Supplementary Fig. S5).

### Higher levels of hexoses and starch are accumulated in mature seeds

To test whether the increased CWI activities resulted in effects on the synthesis of primary metabolites, the contents of sugars, protein, and oil in mature seeds were quantified. Soluble sugars showed a significant increase in levels of hexoses in the RNAi backgrounds, but there were nearly no differences for maltose in comparison with the wild-type control plants (Table 1). Sucrose appeared to be more abundant than hexoses, but its content was not significantly affected in RNAi plants, except for *CIF1-6-1*. Starch, protein, and oil were further analyzed and it was found that more starch and protein were produced in RNAi plants. Interestingly, the percentage of oil was the same in both backgrounds (Table 1). These findings suggest that the hexoses liberated in a CWI-dependent manner during seed maturation and the elevated CWI activities are more closely associated with increases of starch and protein production in soybean mature seeds.

**Table 1.** Effects on the contents of sugars, protein and oil in RNAi plants

**Genotype**	**Glucose**	**Fructose**	**Sucrose**	**Maltose**	**Starch**	**Protein**	**Oil**
HN53	3.47 ± 0.31	0.87 ± 0.06	36.31 ± 4.07	0.80 ± 0.14	3.84 ± 0.04	40.17 ± 0.43	20.54 ± 0.91
*CIF1-6-1*	6.18 ± 0.77***	2.25 ± 0.35***	46.78 ± 2.86***	0.90 ± 0.08	4.59 ± 0.06***	42.71 ± 0.36***	20.41 ± 0.18
*CIF1-3-1*	4.28 ± 0.13***	1.78 ± 0.62**	38.30 ± 1.10	0.90 ± 0.07	4.64 ± 0.18***	42.79 ± 0.31***	20.30 ± 0.17
*CIF1-5-1*	4.95 ± 0.09***	1.88 ± 0.06***	38.35 ± 0.92	0.95 ± 0.25	4.38 ± 0.03***	42.88 ± 0.17***	20.63 ± 0.23
*CIF1-1–2*	3.83 ± 0.06*	1.70 ± 0.52**	36.50 ± 1.41	0.82 ± 0.03	3.90 ± 0.05*	41.92 ± 0.44***	20.12 ± 0.21

Data represent mean ±SE of at least six biological replicates. Valuesare mg g^−1^ fresh weight of samples for sugar quantifications (glucose, fructose, sucrose, and maltose) and percentage (%) for starch, protein, and oil. Asterisks indicate significant differences in comparison with the control as determined by Student’s *t*-test: ***P<0.001, **P<0.01, *P<0.05.

### Identification of CWI genes co-expressed with *GmCIF1* during seed maturation

**Fig. 7.**
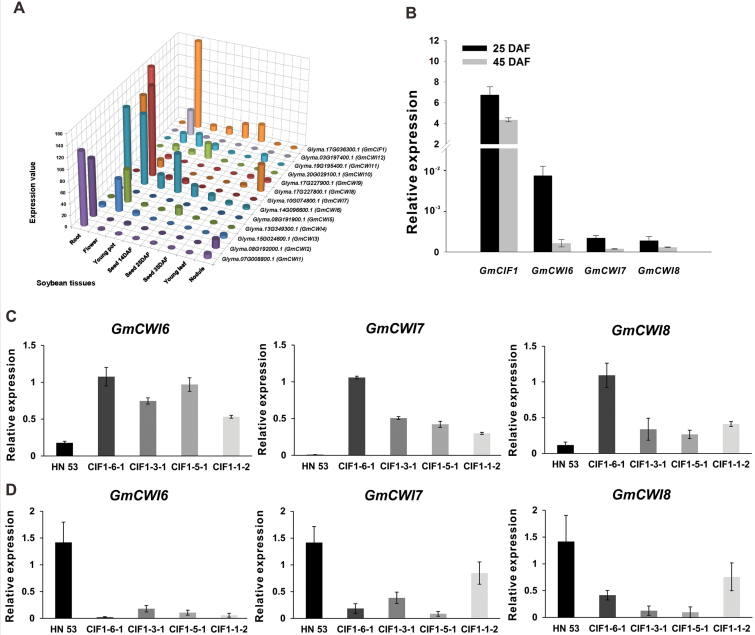
Expression patterns of CWI genes and *GmCIF1* in soybean tissues. (A) RNA-seq expression of putative CWI genes in different tissues. (B) Co-expression of *GmCIF1* and CWI genes in seeds at 25 and 45 d after flowering (DAF) of wild-type plants. (C) CWI gene expression in seed at 25 DAF. (D) CWI gene expression in seed at 45 DAF. The RNA-seq results are given in RPKM (reads per kilobase transcript per million reads) expression values. Data represent mean values ±SE of at least three independent biological replicates for qRT-PCR. *GmACT2/7*, *GmACTII*, *GmEF/αb*, and *GmCYP* were used as reference genes.

The specific modifications of CWI activities at seed maturity stages prompted us to examine whether regulation of CWI mainly depends on post-translational mechanisms. We isolated CWI genes in the soybean genome and analyzed their expressions. BLAST searches from the SoyBase database revealed the presence of 12 putative CWI genes, showing high homologies with *GmCWINV1* (Dimou *et al.*, 2005). Multiple sequence alignment and phylogenetic analyses with other known CWIs and VIs in Arabidopsis and rice suggested that they had well-conserved structures of the CWI gene family (see Supplementary Figs S6 and 7). Based on the initial RNA-seq analysis, six putative CWI genes (*GmCWI3*, *4*, *6*, *7*, *8*, *12*) displayed high transcript levels in flowers and developing seeds (Fig. 7A). Further evaluation by qRT-PCR verified that only three isogenes (*GmCWI6*, *7*, and *8*) were potentially co-expressed with *GmCIF1* at early and mid-stages of seed maturation (25 and 45 DAF) (Fig.7B). In the RNAi backgrounds, expression of CWI genes (*GmCWI6*, *7*, and *8*) increased in seeds at 25 DAF; however, it drastically decreased in seeds at 45 DAF (Fig. 7C, D), which does not relate to the increased CWI activities observed at this developmental stage. Because of low affinity in soybean, the detection of the GmCIF1 protein in the wild-type plants was not successful when using polyclonal antibodies obtained from tobacco, maize, sugar beet, and chicory (data not shown). Overall, identification of novel CWI genes in the soybean genome by examining their tissue-specific expression profiles indicated that at least three CWI isoforms appear to be co-expressed with *GmCIF1* during seed maturation.

## Discussion

### *GmCIF1* plays a functional role as a cell wall invertase inhibitor in soybean

In this study, our primary goal was to isolate and determine the physiological significance of cell wall invertase inhibitor (CIF) genes in post-translational regulation of cell wall invertase (CWI) in soybean plants. Sequence comparisons and phylogenetic analyses revealed that GmCIF1 and GmC/VIF2 share conserved protein structures with other known C/VIFs (Fig. 1). At the same time, functional assays *in vitro* confirmed that GmCIF1 and GmC/VIF2 are indeed invertase inhibitors and, in particular, they showed higher affinities towards CWI than VI (Fig. 2). Consistent with *in silico* predictions of extracellular compartmentation (Supplementary Table S1), ectopic expression of YFP fusion constructs showed that the subcellular localizations of GmCIF1 and GmC/VIF2 are confined to the apoplast in tobacco epidermal cells (Fig. 4), indicating that *GmCIF1* and *GmC/VIF2* may function as extracellular invertase inhibitors *in vivo*.

A tomato CIF, *INVINH1*, was found to show high expression in developing seeds as well as in response to ABA perturbations (Jin *et al.*, 2009). Similarly, *AtCIF1* in Arabidopsis was shown to be an ABA-responsive gene and produced transcript abundances in flowers and seeds (Su *et al.*, 2016). Moreover, our tissue-specific expression analyses determined that *GmCIF1* and *GmC/VIF2* were predominantly expressed in flowers and expression appeared to increase incrementally with seed development (Fig. 3A, C). In addition, both of their transcripts were also significantly induced in response to ABA and ABA-triggered leaf senescence (Fig. 3B, D). The observed predominant expression properties of *GmCIF1* and *GmCIF2* in tissues as well as the response to the phytohormone cue provide evidence of their potential roles in seed development.

Transgenic soybean plants were generated by RNA-mediated silencing of *GmCIF1* in order to investigate its effects on acid invertases *in vivo* (Fig. 5A; see Supplementary Fig. S3A, B). Notably, specific suppression of *GmCIF1* expression resulted in a significant increase of CWI activities in RNAi plants, particularly at late stages of seed maturity, whereas VI activities appeared to be unaffected, suggesting a high specificity of *GmCIF1* against the extracellular invertase (Fig. 5B, C). These results reinforce the view that the functional roles of GmCIF1 are compatible with the *in vitro* effects of the recombinant protein. Finally, expression analyses of several putative CWI genes (Fig. 7A–D) and enzyme activities suggest the significance of GmCIF1 in the control of CWI activities during seed maturation. Taken together, our data provide support for the model that GmCIF1 plays physiological roles as a CIF, which impacts on seed maturation by capping CWI activities.

### Extracellular invertase activities are required for seed development

A large amount of evidence has indicated that CWIs are responsible for sugar partitioning in the apoplast of sinks and that they are involved in the regulation of seed and fruit development in a variety of species (Ruan *et al.*, 2010; Ruan, 2014). Improved seed production has been postulated as a result of elevated extracellular invertase activities in Arabidopsis (Heyer *et al.* 2004). Investigation of a rice extracellular invertase gene, *OsGIF*, revealed that it functions as a determinant of grain filling (Wang *et al.*, 2008). Constant expression of CWIs in maize substantially improved the grain weight and starch content (Li *et al.*, 2013). Similarly, it is not surprising that elevation of CWI activities through silencing or knock-out of CIFs has been shown to bring about improvements in seed weight and production (Jin *et al.*, 2009; Su *et al.*, 2016). These reports suggest an association between functional CWI and sink strength. In agreement with this, the specific suppression of *GmCIF1* expression significantly improved seed weight and production concurrent with markedly elevated CWI activities (Figs 5C and 6A). CWI activities were observed to be higher than VI activities at the mid- and late stages of seed maturity, indicating the fundamental role of CWI, but not VI, in controlling seed development in soybean.

CWI typically catalyzes hydrolysis of sucrose and releases hexoses into the apoplast; hence, the hexoses released are spatially and temporally associated with CWI activities (Roitsch, 1999; Weschke *et al.*, 2003). Starch serves as the major storage form of carbohydrate in developing seeds (Li *et al.*, 2013). After seed germination, the reserved starch degrades into soluble sugars, which fuel cellular respiration to produce energy for seedling growth (Zeeman *et al.*, 2010). Soluble sugars were quantified in mature seeds of the RNAi plants; however, only glucose and fructose were positively affected, reflecting a correlation with the increased CWI activities. Starch content significantly increased in RNAi backgrounds (Table 1). These results suggested that silencing of GmCIF1 may liberate extra CWI activities, which most likely enhance sucrose hydrolysis and starch storage in sinks. Seed protein content statistically increased, whereas the oil content was barely affected in several lines of the RNAi plants (Table 1). These data are in accordance with the fact that seed protein is negatively associated with seed oil content in soybean (Proulx and Naeve, 2009; Eskandari *et al.*, 2013). The improved seed weight and protein content result from the over-accumulation of hexoses, which have been shown to serve not only as nutrients but also as signals to induce storage-associated cell differentiation in seed development (Wobus and Weber, 1999). Moreover, sugar transport from source to sink is one of the major determinants of plant growth (Lemoine *et al.*, 2013), and therefore we hypothesize that the modified sink strength may initially benefit from the improved efficiency of sugar production in source leaves. This notion was supported by the finding of increased CWI activities in sources leaves of RNAi plants (Supplementary Fig. S4D). The increased CWI activities correlate with the increase of photosynthetic capacity in the sources (Dai *et al.*, 1999; Jin *et al.*, 2009). Taken together, the elevation of CWI by the silencing of its inhibitor may facilitate sucrose unloading and release more hexoses, which are required for starch and protein synthesis during seed maturation.

### GmCIF1-mediated release of CWI repression depends on post-translational mechanisms

A previous study showed that NtCIF was co-expressed with CWI in tobacco suspension-cultured cells throughout the entire culture period (Krausgrill *et al.*, 1998). The presence of a CWI inhibitor was demonstrated with spatial and temporal expression coupled with a CWI gene, *Lin5*, in the phloem parenchyma of young fruit (Jin *et al.*, 2009). More recently, co-expression of CWIs and CIFs has been shown in cotton and Arabidopsis during seed development and germination (Wang *et al.*, 2012; Su *et al.*, 2016), suggesting that post-translational regulation of CWI accompanied by the co-expression of CWI genes and their inhibitors is a common phenomenon in plants. To test this view, a number of putative CWI isogenes were identified in the soybean genome based on a search for homologs (see Supplementary Fig.s S6 and S7). Among the 12 identified CWI genes in the soybean genome, three isoforms were shown by the presence of transcripts during seed maturation (25 and 45 DAF) on the basis of expression analysis (Fig.7A, B). Combined with expression patterns of GmCIF1 in the wild-type plants (Figs 3A and 7A, B), these results suggest that at least three CWI genes are potentially co-expressed with *GmCIF1* at the onset of seed maturity. Co-expression of CWI genes and *GmCIF1* provides the potential for the direct interaction of their protein products. Moreover, such co-operation would help the efficient translocation of the hydrolyzed product to sink tissues via fine-tuning of enzyme activities (Ehneß and Roitsch, 1997). However, as concluded in a previous study on maize (Bate *et al.*, 2004), it remains a big challenge to determine which CWI gene(s) would be inhibited by GmCIF1 as more than three possible CWIs targets for GmCIF1 can be envisioned.

Increased invertase activities in sink tissues are probably due to post-translational regulation via the alteration of levels of inhibitor proteins, as reported in a variety of species (Greiner *et al.*, 1999; Jin *et al.*, 2009; Leskow *et al.*, 2016; Su *et al.*, 2016). The increased CWI transcripts were found to be concomitant with elevated CWI activities at early stages of seed maturity (25 DAF) in the RNAi backgrounds (see Supplementary Fig. S4A). Interestingly, in the RNAi backgrounds, decreased but extremely low levels of CWI gene transcripts (*GmCWI6*, *7*, and *8*) were detected at the mid- or late stages of seed maturation (45 and 65 DAF), which were not in concert with the aforementioned significant elevations of CWI activities at these times (Fig. 5B, C). By contrast, *GmCIF1* expression appeared to be more abundant at these stages of seed maturity in the wild-type background (Fig 7A, B). The inconsistency observed between the enzyme activities and gene expressions of CWI supports the idea that, at least during the seed maturation, CWIs are regulated at a post-translational level by GmCIF1. Moreover, along with the significantly elevated CWI activities in the RNAi backgrounds and concurrent with increases of GmCIF1 expression in the wild-type background, the post-translational regulation of CWI correlates more closely with the amount of inhibitor transcripts (or activities), suggesting a pivotal role for GmCIF1 later in the control of primary metabolism and biomass accumulation.

## Conclusions

The search for the primary target of GmCIF1 in combination with enzyme assays and RNAi analysis revealed that GmCIF1 specifically inhibits CWI. We provide evidence that GmCIF1 plays a fundamental role in the post-translational control of extracellular invertase, which results in an impact on seed development and sucrose hydrolysis at a tissue-specific level. Silencing of GmCIF1 significantly elevated CWI activities in soybean plants. Our results are instrumental in helping to decipher how invertase inhibitors regulate CWI during seed maturation. Moreover, unravelling the mechanisms underlying the post-translational modulation of extracellular invertase in soybean has implications for plant biotechnology and may provide new cues for improving crop performance through manipulating the interaction of CWI and its inhibitory proteins. The potential roles of GmCIF1 in resistance to biotic and abiotic stresses as well as its impact on nutrient use efficiency will be the subject of future investigations.

## Supplementary Material

Supplementary Data
