# Supplementary material for: Suppression of extracellular invertase inhibitor gene expression improves seed weight in soybean (Glycine max)
Source: J Exp Bot. 2016 Dec 7;68(3):469–82. doi: 10.1093/jxb/erw425 (PMC5441900; doi:10.1093/jxb/erw425)
Supplement: Supplementary Data [file erw425_Supplementary_Data.zip › supplementary_tables_S1_S2_figures_S1_S7.pdf]

# Supplementary Data

**Table S1.** Predictions in subcellular localization of GmCIF1 and GmC/VIF2

| | Program | Extra | ER | Golgi | CHL | MT | Other |
|---|---|---|---|---|---|---|---|
| GmCIF1 | PSORT | 0.705 | 0.100 | 0.100 | na | na | 0.100 |
| | TargetP | 0.951 | na | na | 0.090 | 0.112 | na |
| | CELLO | 1.687 | 0.054 | 0.545 | 0.468 | 0.832 | 0.632 |
| GmC/VIF2 | PSORT | 0.820 | 0.100 | na | na | na | 0.311 |
| | TargetP | 0.951 | na | na | 0.090 | 0.112 | na |
| | CELLO | 2.751 | 0.039 | 0.159 | 0.639 | 0.431 | 0.210 |

The predictions of protein targeting were performed by the programs of PSORT (http://psort.hgc.jp/form.html), TargetP (http://www.cbs.dtu.dk/services/TargetP/), and CELLO (http://cello.life.nctu.edu.tw/). Extra, Apoplast; ER, endoplasmic reticulum; Golgi, Golgi body; CHL, chloroplast; MT, mitochondria; na, not applicable. The value indicated the probability of localization in the subcellular compartment.

**Table S2.** List of oligonucleotides used for qRT-PCR and cloning

| **Primer Name** | **Comment** | **Primer sequence (5’→3’)** |
|---|---|---|
| **E.coli expression** | | |
| Gm C/VIF2F | C/VIF2_AttB1_TEVFor | TATTTTCAGGGCAGAACCTTGCTTCCAGAAAATG |
| Gm C/VIF2R | C/VIF2_AttB2Rev | AGAAAGCTGGGTTTTAGAGCAACAATCTAACAA |
| GmCIF1F | CIF1_AttB1_TEVFor | TATTTTCAGGGCATTTCAGTTCCAGCAACTAAC |
| GmCIF1R | CIF1_AttB2Rev | AGAAAGCTGGGTTCTAGAGCAATTGTCTAACTA |
| attB1 | attB1_adapter | GGGGACAAGTTTGTACAAAAAAGCAGGCT |
| attB2 | attB2_adapter | GGGGACCACTTTGTACAAGAAAGCTGGGT |
| **qRT-PCR** | | |
| GmC/VIF2qF | C/VIF2_q_For | TTCAATACCATCAAGCCACT |
| GmC/VIF2qR | C/VIF2_q_Rev | TCAGCACAAGAACTCAAGG |
| GmCIF1qF | CIF1_q_For | CTCTGCTGGGAAATCGCCACTCAC |
| GmCIF1qR | CIF1_q_Rev | ATTAAATTGCATCATCCCCTCCGTTG |
| Gm07G237300F | 07G237300For | CATCATGCTGACTTGGTGCC |
| Gm07G237300R | 07G237300Rev | AGCACGTATGGTACTTGTCTCA |
| GmCWI6qF | CWI6_qFor | ACAGAATTGGATGAATGGGCCT |
| GmCWI6qR | CWI6_qRev | GAGCCTGACCAGCAGCTATT |
| GmCWI7qF | CWI7_qFor | TGGATACCATTTTCAACCTCGTAA |
| GmCWI7qR | CWI7_qRev | TCCTTTGATACTGCGTGTCCC |
| GmCWI8qF | CWI8_qFor | TATCACTGCATCACAGGCCG |
| GmCWI8qR | CWI8_qRev | CTGCAGTGTGTTCTGTTTGGT |
| GmEF/bqF | EF/αb_q_For | CCACTGCTGAAGAAGATGATGATG |
| GmEF/bqR | EF/αb_q_Rev | AAGGACAGAAGACTTGCCACTC |
| GmCYPqF | CYP_q_For | ACGACGAAGACGGAGTGG |
| GmCYPqR | CYP_q_Rev | CGACGACGACAGGCTTGG |
| GmACTII_qF | ACTII_q_For | ATCTTGACTGAGCGTGGTTATTCC |
| GmACTII_qR | ACTII_q_Rev | GCTGGTCCTGGCTGTCTCC |

| | | |
|---|---|---|
| GmACT2/7_qF | ACT2/7_q_For | CTTCCCTCAGCACCTTCCAA |
| GmACT2/7_qR | ACT2/7_q_Rev | GGTCCAGCTTTTCACACTCCAT |
| **Co-localization** | | |
| GmC/VIF2_L_F | CIF2_attB1 | GGGGACAAGTTTGTACAAAAAAGCAGGCTCCATG ACAAACTTGAAGCCTC |
| GmC/VIF2_L_R | CIF2_attB2(-ST) | GGGGACCACTTTGTACAAGAAAGCTGGGTCGAGCA ACAATCTAACAATAGC |
| GmCIF1_L_F | CIF1_attB1 | GGGGACAAGTTTGTACAAAAAAGCAGGCTCCATG AAAATTATGGAATCAT |
| GmCIF1_L_R | CIF1_attB2(-ST) | GGGGACCACTTTGTACAAGAAAGCTGGGTCGAGCA ATTGTCTAACTATAG |
| AtCIF1_L_F | AtCIF1_attB1 | GGGGACAAGTTTGTACAAAAAAGCAGGCTCCATG AAGATGATGAAGGTGATGATG |
| AtCIF1_L_R | AtCIF1_attB2 | GGGGACCACTTTGTACAAGAAAGCTGGGTCGAGCA CCAACAAGTTCTTCCTCTATTGAA |
| BvCWI-1_L_F | BvCWI-1_attB1 | GGGGACAAGTTTGTACAAAAAAGCAGGCTCCATGC TATACAAAGGAGTATATC |
| BvCWI-1_L_R | BvCWI-1_attB2 | GGGGACCACTTTGTACAAGAAAGCTGGGTCGAGCA TGCAATGTTGGCTTTCTTCATG |
| **RNAi vector construction** | | |
| GmCIF1_F | BamHI_GmCIF2-F P1 | GGATCCCCGAAGCAGATGTGACAGGG |
| | SmaI_GmCIF2-F R1 | CCCGGGCTAGAGCAATTGTCTAACTA |
| GmCIF1_R | KpnI_GmCIF2-R P1 | GGTACCCTAGAGCAATTGTCTAACTA |
| | SacI_GmCIF2-R R1 | GAGCTCCCGAAGCAGATGTGACAGG |
| FAD2-1b intron | SmaI_FAD2I P1 | TAGTTAGACAATTGCTCTAGCCCGGGCCACTAGGCA TGGTATGATG |
| | KpnI_FAD2I R1 | GGTACCTGTTTCCTTTGCTAGACCCTGTG |

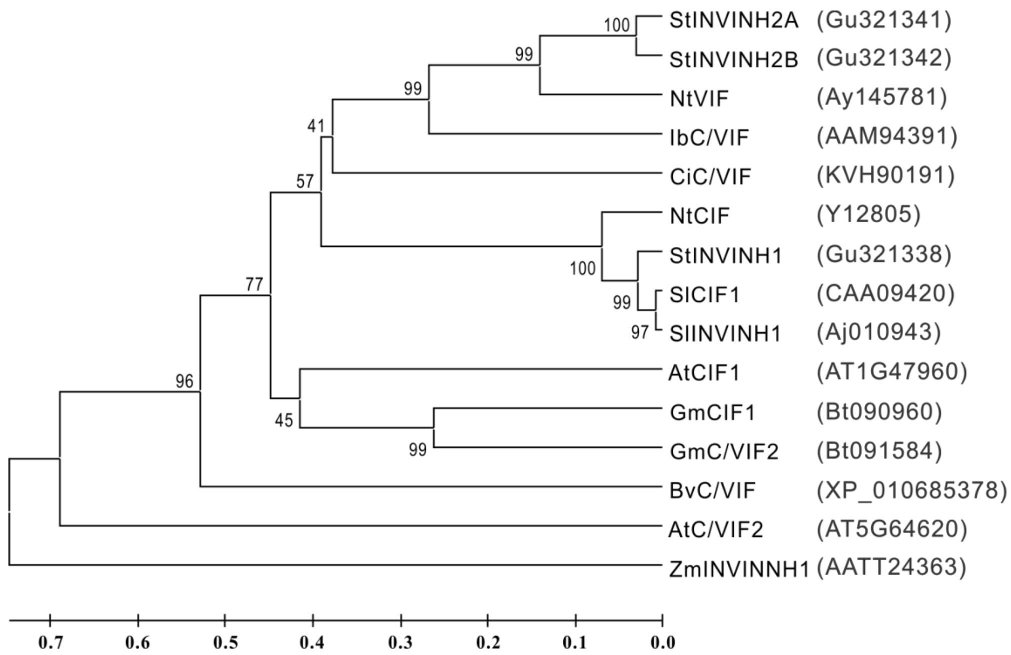


**Figure S1.** Molecular phylogenetic relationship of the function characterized invertase inhibitors. Evolutionary analysis was conducted in MEGA 6.0 (Tamura et al. 2013), using the UPGMA method. The percentage of replicate trees in which the associated taxa clustered together in the bootstrap test (5000 replicates) is shown next to the branches. The analysis involved 15 amino acid sequences of C/VIFs.

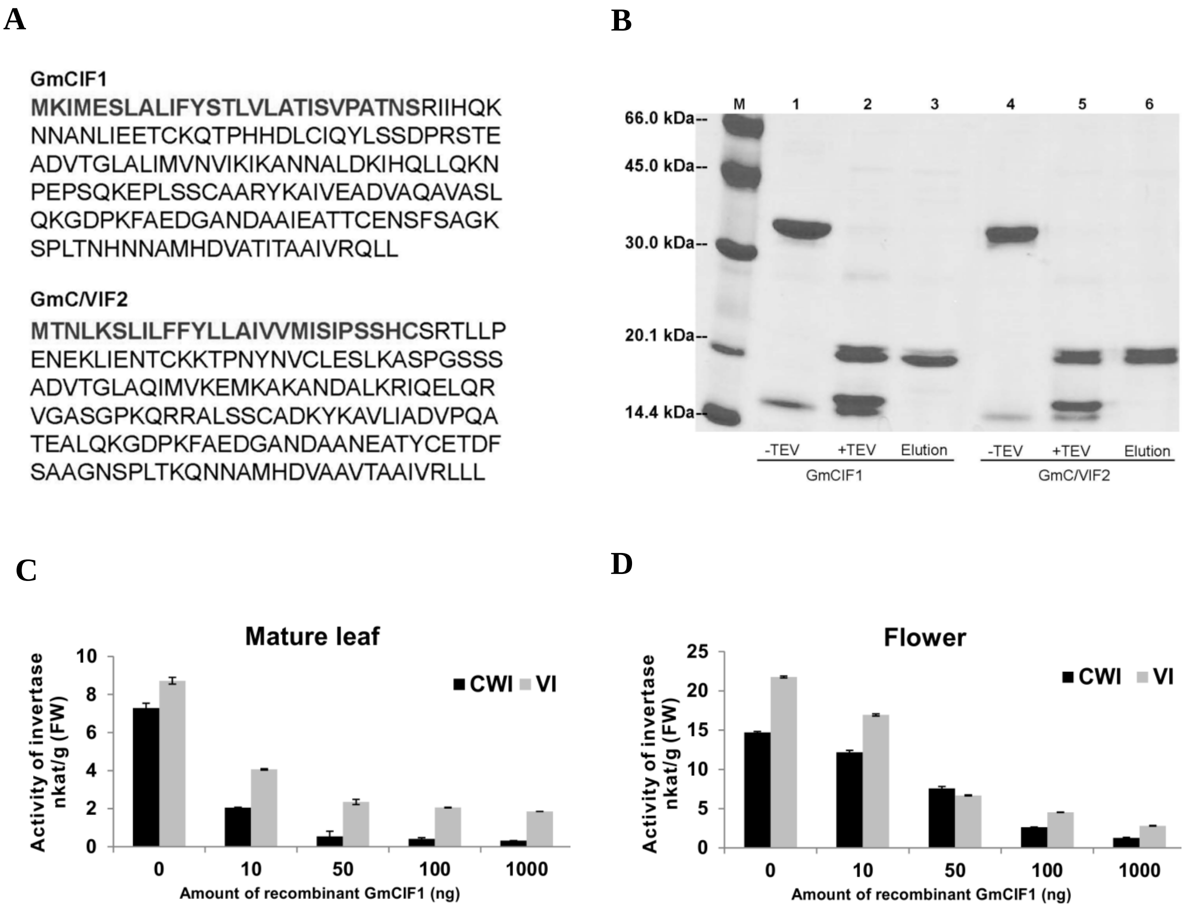


**Figure S2.** Inhibitory effect of the recombinant GmCIF1 and GmC/VIF2. (A) Predicted N-terminal signal peptides were marked in bold. (B) Purification of GmCIF1 and GmC/VIF2. Purified thioredoxinA fusion proteins (lane 1 and 4); purified proteins with cleavage of TEV protease (lane 2 and 5); finally purified GmCIF1 and GmC/VIF2 (flow-through after Ni-TED chromatography) were shown in lane 3 and 6; lane M, low molecular weight markers. Proteins were separated on a 15% SDS-PAGE gel. (C) and (D) The *in vitro* continuous inhibitory effects of GmCIF1 and GmC/VIF2. CWI and VI protein fractions were extracted from mature leaves and flowers.

**A**

ATGAAAATTATGGAATCATTAGCTCTTATCTTCTACAGTACTCTTGTTTTAGCTACGATTTCAGTTCCAGCAACTAA
CTCCAGAATCATCCATCAAAAAAACAATGCCAATCTGATTGAAGAAACTTGCAAGCAGACACCCCATCACGACCT
TTGCATCCAATACCTCTCCTCCGACCCTCGCAGCACCGAAGCAGATGTGACAGGGCTGGCACTTATTATGGTC
AACGTAATCAAAATCAAAGCAAACAATGCATTGGACAAAATCCACCAACTGCTTCAGAAAAACCCTGAACCTA
GTCAAAAGGAACCACTGAGTTCGTGTGCTGCTAGATACAAAGCAATTGTGGAAGCTGACGTGGCACAAGCC
GTTGCGTCTCTGCAGAAAGGAGACCCCAAGTTCGCAGAAGATGGTGCCAATGATGCTGCTATTGAGGCCACC
ACTTGTGAGAACAGCTTCTCTGCTGGGAAATCGCCACTCACCAATCACAACAATGCTATGCACGATGTTGCAA
CCATAACTGCAGCTATAGTTAGACAATTGCTCTAG

**B**

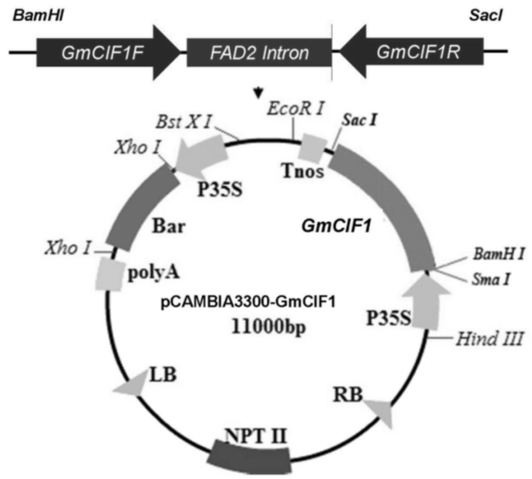


**C**

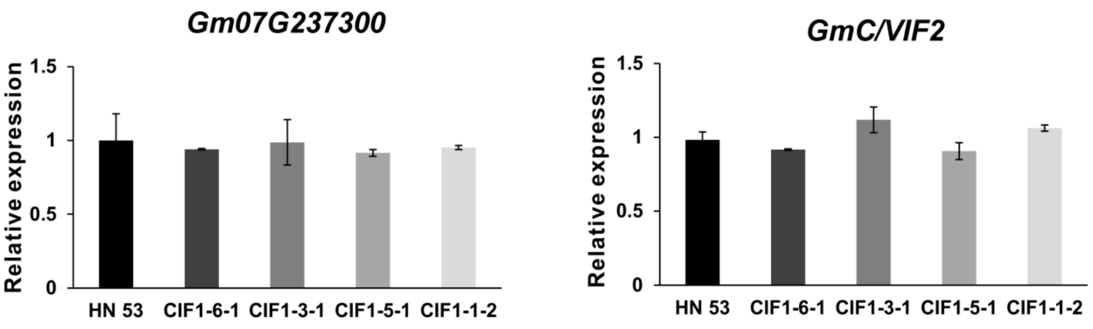


**Figure S3.** Vector construction for the RNAi-mediated silensing of *GmCIF1*. (A), (B) The partial coding sequence (in grey) of *GmCIF1* was used for the vector construction. (C) Expression analysis of GmCIF1 homologs. Three deduced targets sites (underlined) were predicted by the online program siRNA Target Finder (http://www.genscript.com/siRNA_target_finder.html).

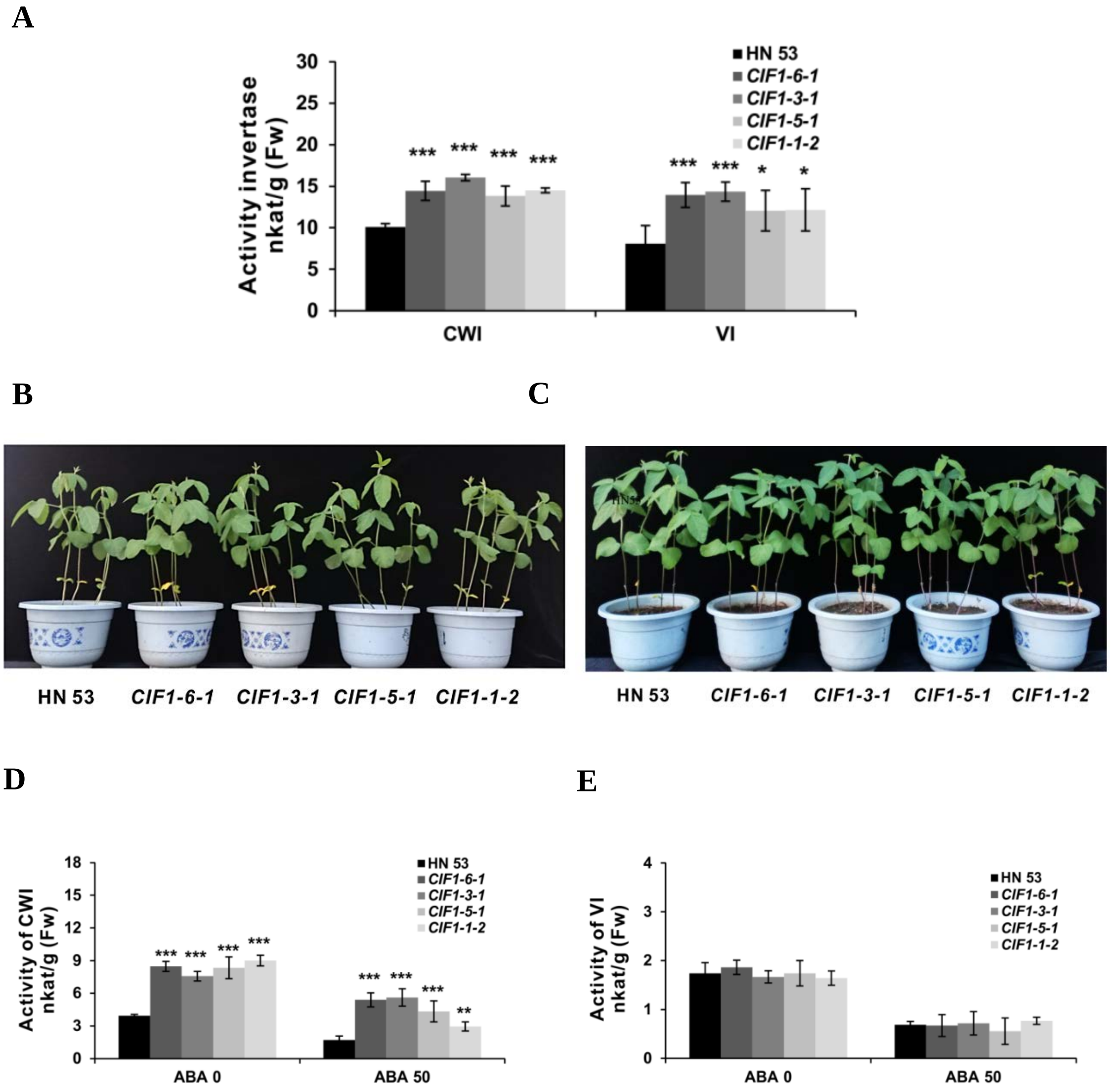


**Figure S4.** Effects of the acid invertase activities and plant growth with/without ABA. (A) Acid invertase activities increased in seeds (25 DAF) of RNAi lines. (B) Plant image before ABA treatment. (C) Plant image after 4 days ABA treatment. (D) Effect of CWI activities. (E) Effect of VI activities. Plant leaves were sprayed with 50 μM ABA for days. Mature leaves were harvested for the acid invertase extraction. The enzyme activity data represent means ± SE of at least four biological replicates and asterisk (*) indicates significant differences in comparison with the control at Student's t-test, *P<0.001* (***), *P<0.05* (*) level.

A

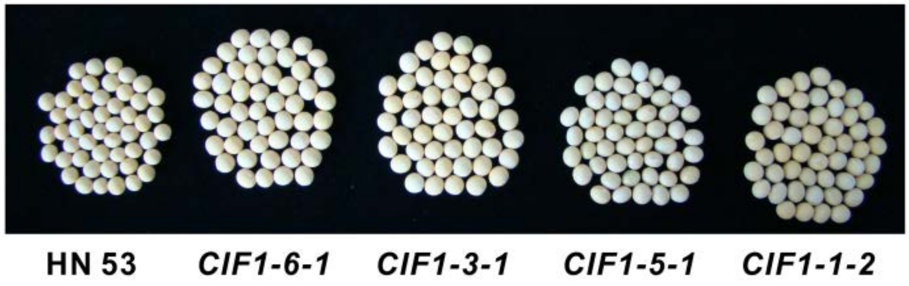


B

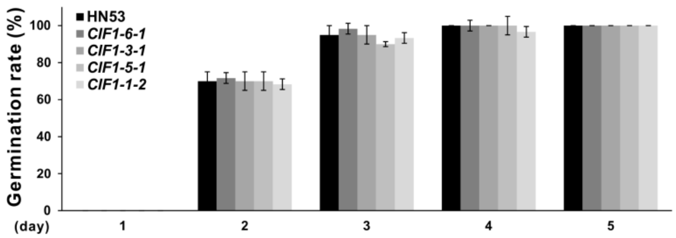


C

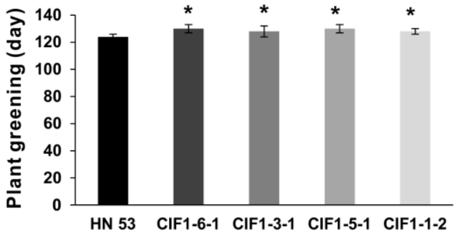


**Figure S5.** Phenotypic effects of the seed weight, germination, and plant senescence. (A) Impact on seed weight (50 seeds). (B) Effect of seed germination. (C) Effect of plant greening. The seed germination (n=30 seeds) and plant senescence (n=40 plants) data represent means ± SE of at least four biological replicates and asterisk (*) indicates significant differences in comparison with the control at Student's t-test, *P<0.0001* (***), *P<0.001*(**), *P<0.01* (*) level.

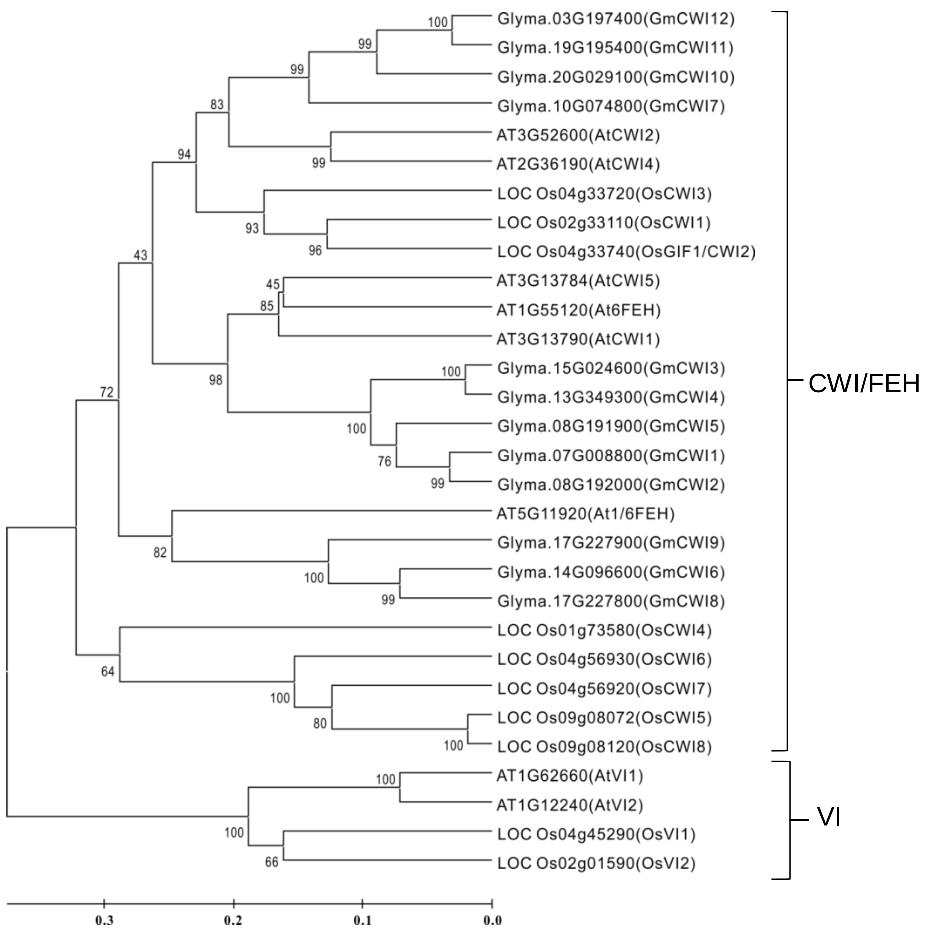


**Figure S6.** Phylogenetic tree of CWIs and VIs in *Arabidopsis*, rice and soybean. The putative soybean CWIs are clustered in subgroup of the CWI family. Evolutionary analysis was conducted in MEGA 6.0, using the UPGMA method. The percentage of replicate trees in which the associated taxa clustered together in the bootstrap test (5000 replicates) is shown next to the branches. The analysis involved 30 amino acid sequences of acid invertases.

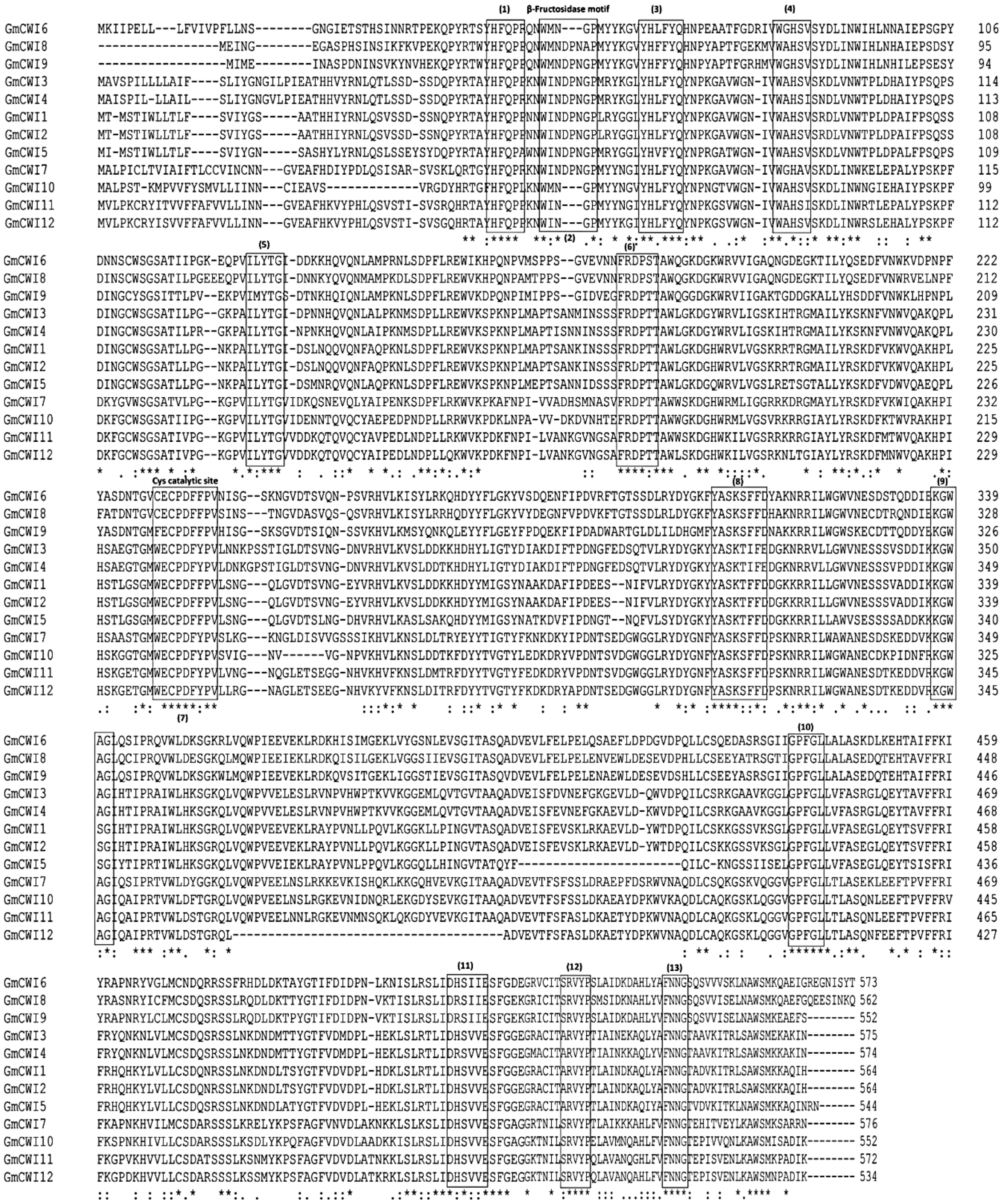


**Figure S7.** Multiple sequence alignment of the cell wall invertase family in soybean. The boxed region indicates the 13 well-conserved regions from the known CWI of the selected green plants.
